# Effect of Maternal Smoking on Breast Milk Interleukin-1α, β-Endorphin, and Leptin Concentrations

**DOI:** 10.1289/ehp.7702

**Published:** 2005-06-15

**Authors:** Vincenzo Zanardo, Silvia Nicolussi, Stefania Cavallin, Daniele Trevisanuto, Angelo Barbato, Diego Faggian, Flaviano Favaro, Mario Plebani

**Affiliations:** 1 Department of Pediatrics and; 2 Institute of Laboratory Medicine, Padua University School of Medicine, Padua, Italy

**Keywords:** β-endorphin, cigarette smoke, colostrum, interleukin-1α, leptin, maternal smoking, transitional milk

## Abstract

Tobacco smoke is immunotoxic, but the effect of smoking on the immunologic function of the mammary gland of mothers who smoke cigarettes (“smoker mothers”) has not been studied. Our objective was to test, in smoker mothers, the colostral and transitional milk concentrations of interleukin-(IL)1α. The immunomodulators β-endorphin and leptin were also tested. Pregnant women who self-identified as smokers (≥ 5 cigarettes per day through pregnancy) or nonsmokers were recruited for study participation. The study population included 42 smoker and 40 non-smoker nursing mothers, with otherwise uncomplicated gestation, delivery, and puerperium, who were breast-feeding *ad libitum* their healthy neonates. Colostrum was obtained on the third postpartum day at 0900 hr and transitional milk on the 10th postpartum day at 0900 hr. IL-1α concentrations were significantly reduced in the colostrum of smoker mothers compared with nonsmoker mothers (*p* < 0.01). Colostral β-endorphin and leptin concentrations were comparable. No significant differences were found between smoker and nonsmoker lactating mothers in transitional milk concentrations of IL-1α, β-endorphin, and leptin. Moreover, β-endorphin and leptin concentrations were significantly reduced in transitional milk samples compared with colostrum of both smoker and nonsmoker mothers (*p* < 0.05); also, IL-1α transitional milk concentrations were reduced compared with colostrum, but without any significance. This analysis shows that maternal smoking alters the colostral milk levels of the proinflammatory cytokine IL-1α. The altered postnatal provision of alternative source of the proinflammatory cytokine IL-1α adds understanding to how breast-feeding could be nonprotective against infections among the neonates nursed by smoker mothers.

Evidence increasingly indicates that breast milk protects against gastrointestinal, respiratory tract, middle ear infections, asthma, and sudden infant death syndrome (SIDS) (Klonoff-Cohen et al. 1995; Kovar et al. 1984; Oddy et al. 2003). Several components of human milk have been postulated to confer this protective effect (Goldman et al. 1998). Breast-feeding might provide an immediate line of defense against infectious agents, which compensates directly for the immaturity of the newborn’s immune system and lowered ability to resist infection. Protection might also be achieved through specific and nonspecific factors in milk, including bioactive enzymes, hormones, growth factors, and immunologic agents that augment and stimulate the development of immature host defense. Thus, it is not clear which of the many components of this complex, changing biologic fluid account for the protective effect.

Passive smoking in the same room as the infant increases the risk for respiratory disease and SIDS (Gordon et al. 2002), whereas breast-feeding is protective for SIDS among nonsmokers, but not smokers, when adjusted for potential confounders (Klonoff-Cohen et al. 1995), raising the possibility that there are also immunologic changes in the breast milk. However, a clear negative effect of nursing by a smoker mother has not been demonstrated (Mitchell et al. 1993), and very little is known of the mechanisms by which smoke might account for the reduced protective effect of breast milk (Blizzard et al. 2003; Elliot et al. 2003).

Cigarette smoke is composed of > 5,000 chemicals, including approximately 70 carcinogens (Stedman 1968), very few of which have actually been assayed for immunosuppressive activity (Ouyang et al. 2000). Conflicting reports exist regarding the effects of nicotine on cytokine production, and little is known about the nature of the immunosuppressive compounds in cigarette smoke (Ouyang et al. 2000). Nevertheless, the major phenolic components of cigarette tar, hydroquinone and catechol, have been reported to suppress the production of interleukin-(IL)1β, IL-2, and interferon-γ (Kalf et al. 1996; Ouyang et al. 2000). Breast-feeding substantially increases absorption of nicotine compared with only environmental tobacco smoke when the mother smokes (Dahlström et al. 1990; Luck and Nau 1985; Schulte-Hobein et al. 1992). In smoker mothers, the milk:plasma concentration ratio of nicotine is 2.9, whereas that of the primary metabolite cotinine is 1.2 (Dahlström et al. 1990). Nicotine has received much attention because it is immunotoxic, triggering the immune system and altering the humoral and cellular immunity and the levels of certain cytokines and their receptors (Sayers and Drucker 1999; Sopori et al. 1998).

It has only recently been shown that human milk contains cytokines (Goldman et al. 1996). Cytokines are small soluble glycoproteins that act in an autocrine or paracrine manner by binding to specific cellular receptors, operating in networks, and orchestrating the immune system’s development and function. Early milk has an abundance of cytokines at a time when neonatal organ systems are immature, suggesting that these bioactive components of milk might be important in neonatal immunity development. Growth, differentiation, and immunoglobulin production by B-cells were among the first activities found in human milk and attributed to the presence of cytokines (Bentzen 1994).

IL-1α is one of the two forms, indistinguishable in their biologic activities, of IL-1, the prototypic proinflammatory cytokine, involved in the mechanisms underlying many infectious and noninfectious inflammatory diseases. Although IL-1 can up-regulate host defenses and function as an immunoadjuvant, it is a highly proinflammatory cytokine. The margin between clinical benefit and unacceptable toxicity in humans is exceedingly narrow. In contrast, agents that reduce the production and/or activity of IL-1 are likely to have an impact on clinical medicine. The synthesis, processing, secretion, and activity of IL-1 are tightly regulated events. For instance, the presence of an IL-1 receptor antagonist is an important mechanism through which IL-1 signaling is down-regulated, and therefore its activity is reduced (Dinarello 1998). IL-1 is probably the first cytokine that has been quantified in human milk (Munoz et al. 1990). IL-1β, but not IL-1α, was frequently investigated and found in colostrum and early milk samples from healthy lactating mothers. To the best of our knowledge, the effect of cigarette smoking on the immunologic function of the mammary gland has not been studied previously.

Our objective was to determine whether there is a relationship between IL 1-α levels in milk and smoking habits in nursing mothers, with the consequent implications on the development of the immune system. Other factors in milk considered for association with infection risk in infancy were the immunomodulators β-endorphin and leptin. β-Endorphin was assessed because it is produced by the epithelial cells (i.e., the primary cells in human milk) (Khachaturian et al. 1958), it exists at detectable levels in human milk (Zanardo et al. 2001), and it influences the behavior of other cells and tissues. Leptin, induced by lipopolysaccharide (LPS) and cytokines, participates in the host response to inflammation by modulating the host immune and cytokine responses after LPS (Faggioni et al. 1999). Thus, any effects of a given cytokine could be tested for specificity by comparison with the other immunologically active components.

## Materials and Methods

Longitudinal and cross-sectional human milk samples were obtained from mothers hospitalized in regional tertiary maternity care at Padua University (Italy), from June to December 2002. Human milk was collected by mothers via commercial breast pumps, frozen immediately, and stored. Collection date and time were recorded, as were infants’ birth date, gestational age, birth weight, and sex and mothers’ demographic and anthropometrical characteristics [age, education, parity, body mass index (BMI)], diagnoses, pregnancy complications, and route of delivery (cesarean section vs. vaginal).

Before the infant’s birth, pregnant women were asked by experienced research midwives to participate in a prospective study. The women received verbal and written information about the aim and design of the study. Only healthy women ≥ 18 years of age, not taking anti-inflammatory medications at the time of enrollment, with otherwise uncomplicated gestation, delivery, and puerperium and who planned to obtain care for their newborns through rooming-in and exclusively breast-feeding their newborns were eligible. Allergy in mothers was not an exclusion criterion because of the findings that maternal atopy shows no relationship with cytokine level in milk (Bottcher et al. 2000). After the women accepted participation, their smoking history was recorded.

Of the 1,217 eligible participants, 25 of 26 self-identified as smokers (≥ 5 cigarettes per day through pregnancy until last trimester) were recruited for study participation. One was excluded from the final analysis because of maternal fever. Control participants included consecutive women without history of smoking and matched a smoking participant on the basis of overall inclusion criteria. A written informed consent was obtained from all participants.

### Milk specimen collection and processing.

Breast milk samples were collected during the third postpartum day (before discharge) and on the 10th postpartum day, close to expression of the colostrum and transitional milk production phases. Collection was standardized to reduce bias and potential diurnal variability in cytokine measurements. Specimens were obtained within 1 hour of the first feeding in the morning, defined as 0800 hr to 0900 hr. Mothers were asked not to feed for 3 hr before collection. All mothers were able to provide milk at each sampling point. An aliquot of 3 mL was taken using a manual breast pump. Samples were temporarily stored at −70°C in sterile plastic tubes and used for assays.

Samples were thawed and centrifuged for 10 min at 1800 rpm at 4°C, after which the lipid layer and cellular elements were removed. The aqueous fraction was filtered (0.45-μm Acrodisc; Gelman Sciences, Ann Arbor, MI, USA) and was used for cytokine, β-endorphin, and leptin determinations, with the lipid layer frozen for other studies.

### Milk IL1-α, β-endorphin, and leptin assays.

Filtered aqueous milk fractions were assayed for IL-1α (by CytElisa Human IL-1α ACCUCYTE; CytImmune Sciences Inc., Rockville, MD, USA), β-endorphin (beta-Endorphin 60 T kit RIA; Nichols Institute Diagnostic, San Juan Capistrano, CA, USA), and leptin (ACTIVE Human Leptin IRMA, DSL-23100; Diagnostic Systems Laboratories Inc., Webster, TX, USA) levels. All fractions were assayed undiluted. The coefficients of variation for intraassay and interassay, respectively, are the following: IL-1α, 8.3 and 11.2%; β-endorphin, 4.1 and 7.1%; and leptin, 3.7 and 6.6%.

### Statistical analysis.

Unless otherwise specified, results are expressed as mean concentrations in nanograms per liter or micrograms per liter ± SE. The *p* = 0.05 significance level was used for the statistical analysis. We grouped human milk samples into third postpartum day smoker and nonsmoker mother groups, with 42 and 40 samples per group, respectively, and into 10th postpartum day smoker and non-smoker mother groups, with 42 and 40 samples per group, respectively.

Because the concentrations of cytokine IL-1, β-endorphin, and leptin were not normally distributed, we performed a paired analysis with the Mann-Whitney *U*-test. We used the Student *t*-test for the analysis of the other data.

### Ethics.

The research protocol was approved by the Hospital Ethical Committee for Human Research of the University of Padua.

## Results

We analyzed 164 milk samples, collected from 82 mothers, 42 smokers, and 40 nonsmokers. Measurable immunoreactivity levels were observed in most colostral (third day) and transitional (10th day) milk samples throughout the collection period (> 90%). The smoker mothers of healthy-term infants and control nonsmoker mothers of healthy-term infants who planned to breast-feed their infants were comparable in their anthropometric characteristics and those of the respective neonates. The birth weight of the newborn infants of smoker mothers was not significantly lower (Table 1).

IL-1α concentrations were significantly reduced in the colostrum of smoker compared with the nonsmoker control mothers. β-Endorphin and leptin colostrum concentrations were comparable (Table 2).

Moreover, β-endorphin concentrations were significantly reduced in transitional milk samples compared with colostrum of both smoker and nonsmoker mothers. Also, leptin concentrations were significantly reduced in transitional milk samples compared with colostrum of both smoker and nonsmoker group. IL-1α concentrations were lower in transitional milk than in colostrum samples of smoker and nonsmoker mothers, but without any significance (Table 2).

We found no significant differences between smoker and nonsmoker lactating mothers in transitional milk concentrations of IL-1α, β-endorphin, and leptin (Table 2). And we found no correlations between reduced IL-1α levels of the colostral milk samples and related immunomodulator β-endorphin and leptin levels.

## Discussion

In this article, we present novel evidence suggesting that exposure to tobacco smoke during pregnancy may affect the development of the immunologic function of the mammary gland, significantly influencing colostral milk provision of the proinflammatory cytokine IL-1α for breast-feeding infants. By contrast, the concentration of the immunomodulatory factors β-endorphin and leptin were unaffected. In this context, the possibility that delivery modalities, parity, or BMI affects the levels of cytokines and immunomediators in the breast milk is open to speculation.

The identification of reduced IL-1α levels in the colostral milk of mothers exposed to tobacco smoke during pregnancy offers support to the hypothesis that some clinical consequences of smoking on infancy might be initiated and enhanced by altered levels of inflammatory cytokines (Froen et al. 2000; Sayers and Drucker 1999).

Early breast milk may be of particular importance in the development of the mammalian newborn innate and acquired immunity (Goldman et al. 1998). Colostrum has been reported to contain a large amount of cytokines, but little is known as to their concentrations and associations within breast milk (Laiho et al. 2003). IL-1 is probably the first cytokine that has been quantified in human milk (Garofalo and Goldman 1998; Munoz et al. 1990). The synthesis, processing, secretion, and activity of IL-1 are tightly regulated events. For instance, the presence of an IL-1 receptor antagonist is an important mechanism through which IL-1 signaling is down-regulated, and therefore its activity is reduced (Dinarello 1997). Of interest, IL-1β receptor antagonists have been measured in human breast milk (Buescher and Malinowska 1996). To what extent the biologic effect of the cytokine in milk is modulated by the presence of soluble receptors and other cytokine antagonist is open to speculation.

Smoking is a major risk factor for both infantile infections and SIDS. Such infections, both viral and bacterial, also increase the SIDS risk (Gordon et al. 2002; Guntheroth 1989). In experimental animals, nicotine has been shown to depress both the primary and secondary immune response of the lungs, lymph nodes, and spleen (Sayers and Drucker 1999). Interestingly, most studies have shown a decrease in proinflammatory cytokine production (IL-1α, IL-6, and tumor necrosis factor-α) by lung macrophages in smokers, accounting for the greater concentration of tobacco in the lungs (Froen et al. 2000; Sayers and Drucker 1999). These cells have proven very sensitive to both smoking history and the duration of the smoke-free period (Zeidel et al. 2002). Similarly, the greater concentration of tobacco components reported in the breast milk of smoker mothers could imply a different magnitude of immune impairment of the mammary gland macrophages (Klonoff-Cohen et al. 1995). The possibility that altered levels of cytokines and other biologic factors in breast milk modify the immune function in the nursed infants is a very attractive hypothesis (Jones and Warner 2000). Physiologic delays or imbalance in the production of immune factors can occur in mammals, and such delays increase the risk of infection and SIDS (Laiho et al. 2003).

Human milk is an important source of other bioactive substances, including hormones, growth factors, and immunologic factors such as cytokines, but the functional consequences of an overexpression or of a down-regulation of most milk immunomodulatory constituents in neonates are unknown. In our study, the levels of β-endorphin and leptin in breast milk were not related to smoking habits of nursing mothers. The lack of additive effects and the dissimilarity in response to tobacco smoke might indicate that these immunomodulatory molecules are involved in different pathways. In this context, it is interesting that nicotine can cause a release of β-endorphin in various brain regions, and that cytokines and endotoxin can cause a release of β-endorphin in different tissues. Moreover, in mice, leptin, induced by LPS and cytokines, participates in the host response to inflammation by modulating the host immune and cytokine responses (Faggioni et al. 1999).

There are certain limitations in our work. First, only colostral and transitional milk samples were collected. It is well established that milk composition differs with delivery modalities, increasing parturition, throughout the day, at each feeding, and with time. Generally, milk proteins that are produced and secreted in the mammary gland are expected to constitute the largest proportion of protein in human milk and to have more postpartum variation than do serum proteins that are passively transferred into milk (Londerdal and Atkinson 1995). Having multiple cytokine and immunomediators measures taken over time from breast-feeding mothers with a dose–response relationship between smoking levels and levels of agents would permit verification of the assumption underlying this analysis. In addition, the selection of IL-1, of leptin, and of β-endorphin, among the wide range of immunomodulatory factors present in breast milk and interconnecting the neuroendocrine and immune systems, has been made in the context of the present study mainly for convenience and not according to solid scientific reasons.

However, the study has several strengths. Our measure of cytokines in colostrum has been used in previous studies and is valid. Furthermore, most of the colostral and transitional milk samples (> 90%) in the study contained IL-1α, so this is a large enough group to be confident of the results and of measurable presence of IL-1α in the early milk. Nevertheless, it is premature to know how the findings will pertain to later infections, because most children of smoking mothers do not develop infections in the early perinatal time.

## Conclusion

Our data support the suggestion that some clinical consequences of smoking might be initiated and enhanced by the production of inflammatory cytokines from colostrum. Reduced colostral IL-1α concentrations provide additional data to the delineation of the pathophysiologic mechanism that includes mammary gland immunologic dysfunction in the cascade of events that can lead to infections and SIDS. To our knowledge, this is the first study on the effects of smoking on the content of cytokines and immunomediators in human milk. Prospective studies are required to thoroughly assess the differences in the composition of breast milk from mothers with different smoking histories and the immunologic consequences of this for the offspring.

## Correction

The name of co-author Angelo Barbato was added after publication online.

## Figures and Tables

**Table 1:** Demographics, pregnancy, and birth outcomes characteristics of the study groups.

Characteristic	Smokers (*n* = 42)	Nonsmokers (*n* = 40)
Maternal data		
Maternal age (years)	31.1 ± 0.9	31.2 ± 1.1
Maternal smoking (cigarettes/day)	3.2 ± 0.7	
Maternal education (years)	10.5 ± 2	10.6 ± 3
Parity [primiparous (%)]	35.1	41.4
BMI (kg/m^2^)	25.3 ± 4.0	25.7 ± 4.2
Vaginosis (%)	2.3	2
Labor induction (%)	7.1	7.2
Duration of labor (hr)	8.9 ± 4.2	8.6 ± 4.3
Mode of delivery [vaginal (%)]	88	92
Birth data		
Gestational age (weeks)	39.0 ± 0.3	40.0 ± 0.3
Birth weight (kg)	3.23 ± 0.14	3.48 ± 0.09
Sex (%male)	44	42
Apgar score		
1st min	8.8 ± 0.4	9.2 ± 0.2
5th min	9.5 ± 0.1	9.8 ± 0.1

Data are presented as mean ± SD unless otherwise indicated.

**Table 2:** IL-1α, β-endorphin, and leptin concentrations in the colostrum and transitional milk of smoker compared with nonsmoker mothers.

	Colostrum		Transitional milk		
	Smoker^a^	Nonsmoker^b^	Smoker^c^	Nonsmoker^d^	*p*-Value
IL-1α (ng/L)	17.2 ± 4.0	38.4 ± 7.4	14.4 ± 5.2	21.7 ± 12.5	*a* vs. b*
β-Endorphin (ng/L)	353.5 ± 29.1	317.5 ± 27.6	152.8 ± 26.5	127.7 ± 2.3	a vs. c** b vs. d**
Leptin (μg/L)	1.6 ± 0.3	1.5 ± 0.2	0.7 ± 0.1	0.5 ± 0.1	a vs. c* b vs. d**

Data are presented as mean ± SE, or p-value from Mann-Whitney U-test.

* p < 0.05

** p < 0.01.
